# The destructive effect of botulinum neurotoxins on the SNARE protein: SNAP-25 and synaptic membrane fusion

**DOI:** 10.7717/peerj.1065

**Published:** 2015-06-30

**Authors:** Bin Lu

**Affiliations:** Center for Membrane Biology, University of Virginia, Charlottesville, VA, USA

**Keywords:** SNAP-25, Botulinum neurotoxin (BoNT), FRET, EPR, Membrane fusion

## Abstract

Synaptic exocytosis requires the assembly of syntaxin 1A and SNAP-25 on the plasma membrane and synaptobrevin 2 (VAMP2) on the vesicular membrane to bridge the two opposite membranes. It is believed that the three SNARE proteins assemble in steps along the dynamic assembly pathway. The C-terminus of SNAP-25 is known to be the target of botulinum neurotoxins (BoNT/A and BoNT/E) that block neurotransmitters release *in vivo*. In this study, we employed electron paramagnetic resonance (EPR) spectroscopy to investigate the conformation of the SNAP-25 C-terminus in binary and ternary SNARE complexes. The fluorescence lipid mixing assay shows that the C-terminal of SNAP-25 is essential for membrane fusion, and that the truncated SNAP-25 mutants cleaved by BoNT/A and BoNT/E display different inhibition effects on membrane fusion: SNAP-25E (Δ26) abolishes the fusion activity of the SNARE complex, while SNAP-25A (Δ9) loses most of its function, although it can still form a SDS-resistant SNARE complex as the wild-type SNAP-25. CW-EPR spectra validate the unstable structures of the SNARE complex formed by SNAP-25 mutants. We propose that the truncated SNAP-25 mutants will disrupt the assembly of the SNARE core complex, and then inhibit the synaptic membrane fusion accordingly.

## Introduction

The assembly of target SNAREs (t-SNAREs), syntaxin 1A and SNAP-25, on the plasma membrane with the vesicular SNARE (v-SNARE), synaptobrevin 2 (VAMP2), is crucial for Ca^2+^-triggered regulated exocytosis (Poirier et al., 1998; Sollner et al., 1993; Sutton et al., 1998; Weber et al., 1998). The highly-tight ternary SNARE complex is formed at the late step of membrane fusion, and bridges the vesicle and plasma membranes (Pobbati, Stein & Fasshauer, 2006). The SNARE assembly pathway preceding the formation of the ternary complex, however, is not very clear. One study suggests that the three SNARE proteins do not assemble into any intermediate complex before the activation of exocytosis (Lang et al., 2002). Other studies show that t-SNARE proteins may form the binary complex first and then engage with VAMP2 to form the ternary complex (An & Almers, 2004; Hu et al., 2002; Sorensen et al., 2006). However, the composition of the binary complex may differ. Syntaxin 1A may interact with SNAP-25 at a 1:1 or 2:1 molar ratio *in vitro* (Fasshauer & Margittai, 2004). In the 2:1 configuration, VAMP2 may displace the excess syntaxin 1A from the binary complex due to a high affinity with syntaxin 1A/SNAP-25. If not, the 2:1 binary complex is the “dead-end” intermediate on the pathway to membrane fusion (Rizo, 2008; Weninger et al., 2008).

SNAP-25 has two SNARE motifs, designated SN1 and SN2. The C-terminus of SNAP-25 SN2 is known to be the target of botulinum neurotoxins A and E (BoNT/A and BoNT/E), which block the release of neurotransmitters *in vivo* (Binz et al., 1994; Blasi et al., 1993; Schiavo et al., 1993). BoNT/A cleaves nine amino acids from the C-terminus of SNAP-25 between Gln^197^ and Arg^198^, and BoNT/E cuts 26 amino acids from the C-terminus of SNAP-25 at Arg^180^ and Ile^181^. Previous *in vivo* studies (Hunt et al., 1994; Sweeney et al., 1995) have showed that vesicle docking still occurred and unfused vesicles accumulated at the plasma membrane when syntaxin 1A and VAMP2 were inactivated by toxins in neuronal synapses due to the interaction between the calcium sensor synaptotagmin and SNAP-25 (Schiavo et al., 1997). In contract to SNAP-25 in the complex with syntaxin 1A and VAMP2 (Hayashi et al., 1994), the binary complex SNAP-25/synaptotagmin is accessible to both BoNT/A and BoNT/E and the resulting cleavage of SNAP-25 can still bound to synaptotagmin. In PC12 cells, it was found that BoNT/A inhibition could be reversed by elevated Ca^2+^ concentration, but this does not occur in the case of BoNT/E (Gerona et al., 2000). These results suggest that synaptotagmin could be incorporated into synapse vesicle docking (de Wit et al., 2009), and highlight the essential role of the C-terminus of SNAP-25 in Ca^2+^-dependent interactions between synaptotagmin and the SNARE core complex at the late step in regulated exocytosis.

In this study, we employ the pull-down method to prepare the spin-labeled binary and ternary SNARE complex, and investigate the conformation of the C-terminus of SNAP-25 SN1 and SN2 in the SNARE complex assembly pathway. We also investigate the function of the C-terminus of SNAP-25 in the SNARE-mediated membrane fusion by the fluorescence lipid mixing assay involved with different SNAP-25 mutants. We find that the C-terminus of SNAP-25 SN2 is essential for SNARE-reconstituted proteoliposome fusion. Also, although the truncated SNAP-25A (Δ9) can form a SDS-resistant complex with syntaxin 1A and VAMP2, like the wild-type SNAP-25, the fusion activity decreases significantly; while SNAP-25E (Δ26) mutant totally abolishes the fusion activity in accordance with the missing SDS-resistant ability. CW-EPR spectra also display the local conformational changes when SNAP-25 mutants interact with syntaxin 1A and VAMP2 in binary and ternary SNARE complexes which structurally validate the results of functional assays and indicate the key role of tight SNARE core complex formation in synaptic membrane fusion.

## Materials & Methods

### Plasmid construction and site-directed mutagenesis

Full-length syntaxin 1A (amino acids 1–288) was inserted into pET-28b vector to make N-terminal His_6_-tagged protein. Full-length and soluble VAMP2 (amino acids 1–116 and 1–94) were inserted into pGEX-KG vector to make N-terminal glutathione *S*-transferase (GST) fusion proteins. Recombinant syntaxin 1A without the Habc domain (STX1A HT, amino acids 168–288) and wild-type SNAP-25, truncated SNAP-25A (Δ9) and SNAP-25E (Δ26) (amino acids 1–206, 1–197 and 1–180, respectively) were also made as GST fusion proteins. To introduce unique cysteine at the specific position of SNAP-25 and syntaxin 1A, the native cysteines C85, 88, 90 and 92 in SNAP-25 and C145, 271 and 272 in syntaxin 1A were changed to alanines. All cysteine mutants were generated by QuikChange site-directed mutagenesis kit (Stratagene, Cedar Creek, Texas, USA), and were confirmed by sequencing.

### Protein expression, purification, and spin labeling

GST fusion proteins were expressed in *E. coli* Rosetta (DE3) pLysS (Novagene, Darmstadt, Germany) and purified by glutathione-agarose beads (Sigma, Munich, Germany). The cells were grown at 37 °C in LB with glucose (2 g/liter), ampicillin (100 µg/ml), and chloramphenicol (50 µg/ml) until A_600_ reached 0.6–0.8. After adding 0.3 mM isopropylthio-*β*-D-galactopyranoside (IPTG), the cells were further grown for 6 h at 22 °C for VAMP2 and SNAP-25 but at 16 °C for syntaxin 1A. GST fusion protein purification was performed following the procedure described in elsewhere (Lu, Song & Shin, 2010). We add 1% *n*-octyl-glucoside (OG) in the cleavage buffer for syntaxin 1A and VAMP2.

His_6_-tagged syntaxin 1A were expressed in *E. coli* BL21(DE3) CodonPlus-RIL (Stratagene) and purified using Ni-NTA resin (Qiagen, Hilden, Germany). The cells were grown at 37 °C in LB with glucose (2 g/liter), kanamycin (34 µg/ml), and chloramphenicol (50 µg/ml) until A_600_ reached 0.6–0.8. Protein expression was induced by 0.5 mM IPTG, and the cells were grown for an additional 4–6 h at 16 °C. His_6_-tagged protein purification was performed following the procedure described in elsewhere (Lu et al., 2014). Finally, the protein was eluted from the Ni-NTA resin by elution buffer (25 mM HEPES, 100 mM KCl with 250 mM imidazole, pH 7.4).

The cysteine mutants of SNAP-25 and syntaxin 1A were reacted with (1-oxyl-2,2,5,5-tetramethylpyrrolinyl-3-methyl) methanethiosulfonate (MTSSL) spin label at 4 °C overnight while the protein was bound to the GST-agarose beads. To remove free spin label, the beads with bound proteins were extensively washed with the cleavage buffer then cleaved by thrombin (Sigma, Munich, Germany). The spin-labeling efficiency was determined by the 50 µM 2,2,6,6-tetramethyl-4-piperidine *N*-oxide (TEMPO) standard. For all samples, the efficiency was ∼80%.

### Pull-down method preparing for the binary and ternary complex

Details of SNARE complex formation by pull-down method have been described (Lu, Song & Shin, 2010). Briefly, purified His_6_-tagged syntaxin 1A was first added to the Ni-NTA resin solution and incubated for 1 h at room temperature. After washing out the free proteins, 2-fold excess of purified GST-SNAP-25 or 2-fold excess of GST-SNAP-25 and 4-fold excess of soluble VAMP2 were mixed with His_6_-syntaxin 1A to form the t-SNAREs binary complex or *trans*-SNARE ternary complex, respectively. The mixture was incubated at 4 °C overnight. After extensive washing to remove the unbound proteins, the complex was eluted with a buffer containing 250 mM imidazole and 1% *n*-octyl-glucoside (OG). The formation of SNARE complex was confirmed with SDS-PAGE gel.

### Vesicle preparation and membrane reconstitution

Large unilamellar vesicles (LUVs) of 100 nm diameter were prepared using an extruder as described previously (Lu, Song & Shin, 2010). The lipid-to-protein molar ratio was 200:1 (except as noted). For the lipid mixing assay, the mixture of POPC and DOPS in a molar ratio of 85:15 was used. Additionally, NBD-PS and rhodamine-PE (1.5 mol% each) were added to the v-SNARE vesicles for fluorescence detection of lipid mixing. The t-SNAREs were preformed by mixing syntaxin1A HT and SNAP-25 in a molar ratio of 1:1 at room temperature for 60 min before reconstitution. In all cases, proteins were reconstituted by using the dialysis method as described previously (Lu et al., 2014).

### Fluorescence lipid mixing assay

The v-SNARE vesicles were mixed with the t-SNARE vesicles in a molar ratio of 1:9 for the total lipid mixing assay. The final solution for each reaction contained about 1 mM lipids in HEPES buffer (25 mM HEPES, 100 mM KCl, pH 7.4) with a total volume of 100 µl. Fluorescence intensity was measured at the NBD’s excitation and emission wavelengths of 465 and 530 nm, respectively. Fluorescence signals were recorded by a Varian Cary Eclipse model fluorescence spectrophotometer using a quartz cell of 100 µl with 2 mm path length. After 3,600 s, 0.1% (vol/vol) reduced Trion X-100 (Sigma, Munich, Germany) was added to obtain the maximum fluorescence intensity (MFI). All measurements were performed at 35 °C.

### EPR data collection

EPR spectra were obtained using a Bruker ESP 300 spectrometer (Bruker, Bremen, Germany) equipped with a low noise microwave amplifier (Miteq, Hauppauge, New York, USA) and a loop-gap resonator (Medical Advances, Milwaukee, Wisconsin, USA). The modulation amplitude was set to be no greater than one-fourth of the line width. The spectra were collected at room temperature in the first-derivative mode with 1 mW microwave power. The detailed EPR samples preparation followed the methods as described (Lu et al., 2014; Lu, Song & Shin, 2010).

## Results

### The conformation of the C-terminus of SNAP-25 SN1 and SN2

**Figure 1:**
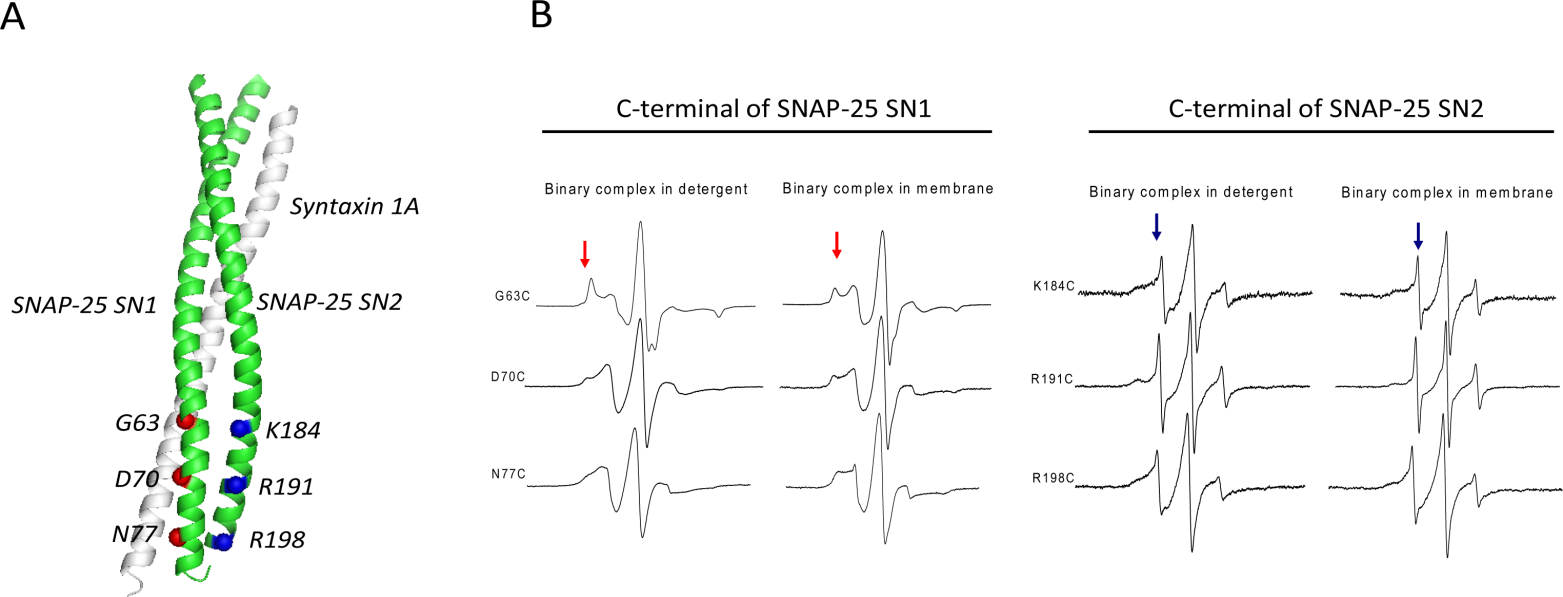
EPR spectra of the C-terminal of SNAP-25 SN1 and SN2 in t-SNAREs binary complex under room temperature. (A) The diagram of the spin-labeled SNAP-25 SN1 and SN2 with syntaxin 1A SNARE motif in t-SNAREs binary complex. The corresponding labeled positions of SNAP-25 SN1 are in red and that of SN2 are in blue. (B) EPR spectra of the spin-labeled C-terminal of SNAP-25 SN1 and SN2 in the full-length His-tag syntaxin 1A and spin-labeled GST-tag SNAP-25 binary complex. EPR spectra of the binary complex in the detergent and in the membrane are shown. The immobile and mobile components are indicated in red and blue arrows, respectively.

To compare the SN1 and SN2 conformations in the t-SNAREs binary complex, we employed site-directed spin labeling (SDSL) and continuous wave (CW) EPR spectroscopy and prepared three cysteine mutants located in the C-terminus of SN1 and SN2, respectively (Fig. 1A): C63, 70 and 77 of SNAP-25 SN1, and C184, 191 and 198 of SNAP-25 SN2. The selected residues were all at the predicted “g” position in the heptad repeats of SNAP-25 SNARE motifs (Poirier et al., 1998). In order to avoid the oligomerization of syntaxin 1A, we used His_6_-tagged full-length syntaxin 1A to pull down the spin-labeled GST-tag SNAP-25. Two-fold excess of purified SNAP-25 were incubated with syntaxin 1A, and then washed extensively to get rid of the free SNAP-25.

The EPR spectra (Fig. 1B) clearly showed that each position in the C-terminal of SN1 had a broad lineshape, especially when the complex reconstituted in the membrane. That indicated that these positions were all interacting with syntaxin 1A, forming the ordered binary complex. However, at the corresponding positions located in the C-terminal of SN2, the EPR lineshape became much sharper, which indicated that these positions may not be completely involved in the binary complex. Based on these data, we propose that SN1 of SNAP-25 and syntaxin 1A form a highly ordered structure, while the C-terminal of SN2 is unstructured. This kind of binary complex is much closer to the active t-SNAREs binary complex *in vivo*, which has 1:1 composition of syntaxin 1A and SNAP-25 (An & Almers, 2004).

**Figure 2:**
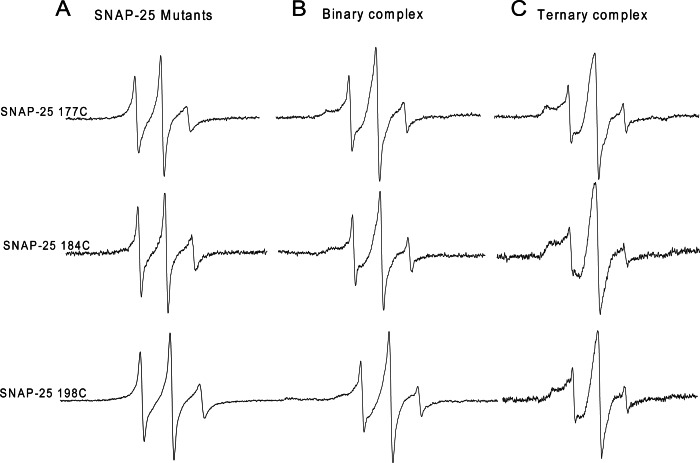
EPR spectra of the spin-labeled C-terminal of SNAP-25 SN2 in binary and ternary SNARE complexes. (A) EPR spectra for three mutants at the C-terminal of SNAP-25 SN2 (177C, 184C and 198C) in solution. (B) EPR spectra for the same spin-labeled positions in membrane-bound syntaxin 1A and SNAP-25 binary complex. (C) Syntaxin 1A-SNAP-25-souble VAMP2 (1-94) ternary complex. All the binary and ternary complexes are prepared by His-tag syntaxin 1A pulling down spin-labeled GST-tag SNAP-25 mutants in the absence or presence of GST-tag soluble VAMP2.

We further investigated the C-terminal conformation of SNAP-25 SN2 in the ternary SNARE complex. His_6_-tagged syntaxin 1A was incubated with two-fold excess of spin-labeled GST-tag SNAP-25 and four-fold excess of GST-tag soluble VAMP2. At three C-terminal positions (C177, 184 and 198), when adding soluble VAMP2, all the EPR spectra became broader, indicating that the C-terminal of SNAP-25 SN2 interacted with VAMP2 and formed the *trans*-SNARE ternary complex (Fig. 2). However, we still detected some sharp components at these positions, which may be because the *trans*-SNARE complex easily splays out at the end of the C-terminus. This also implies that the complete SNARE assembly may require other regulatory proteins (such as synaptotagmin and complexin) at the late zippering step to promote the tight SNARE bundle formation, which is crucial for fusion pore opening.

### The C-terminus of SNAP-25 is essential for synaptic membrane fusion

**Figure 3:**
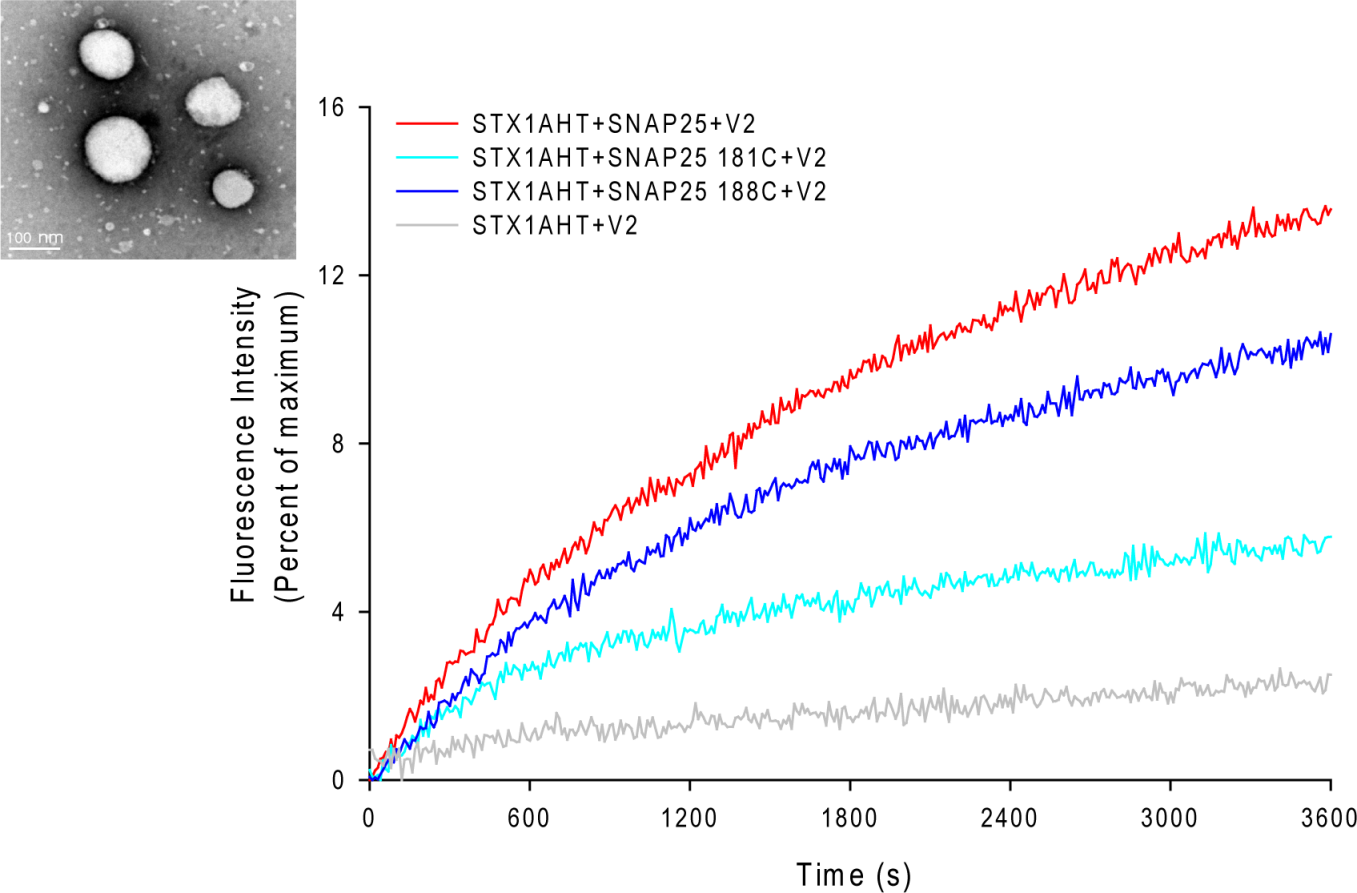
Lipid mixing assay for the nitroxide spin-labeled of SNAP-25 mutants. The curves represent the lipid mixing when the wild-type or nitroxide spin-labeled SNAP-25 at positions of 181 and 188 were used in the proteoliposome fusion assay (protein/lipid ratio 1:200). The data were normalized against the maximum fluorescence intensity (MFI) obtained by adding 0.1% reduced Triton-X-100. The control runs with the v-SNARE vesicles and the t-SNARE vesicles without SNAP-25 (grey curve). The inset in the left corner is the electron micrograph of the SNAREs-reconstituted vesicles used in assays. The vesicles were stained with 1% phosphotungstic acid on the carbon grids. The size of the vesicles was 90 ± 15 nm.

The EPR lineshape analysis revealed that the C-terminal of SNAP-25 SN2 in the binary complex is partially free, and that the sharp components can be detected even in the ternary complex. One might then wonder whether the formation of the completely assembled SNARE complex is required for membrane fusion. To address this question, we examined the fusion activity of the nitroxide spin-labeled SNAP-25 using the fluorescence lipid mixing assay. The SNAREs reconstituted vesicles that were employed in the study are shown in Fig. 3. The nitroxide side chain was relatively bulky, similar in size to that of tryptophan. Therefore, if the formation of the completed coiled coil is necessary for membrane fusion, the alterations at the internal positions (‘a’ or ‘d’) (Poirier et al., 1998) might cause some serious perturbations. As Fig. 3 shows, we detected the distinct lipid mixing activity decreasing with the introduction of the mutations, as compared to the wild-type SNAP-25. Moreover, when the spin-labeled position was closer to the central of SNAP-25 SNARE motif, the perturbation was stronger. SNAP-25 C188 showed a 78% fusion activity of wild-type SNAP-25, while SNAP-25 C181 was just left of 40%. Therefore, our data confirm that SN2 of SNAP-25 is required for activating membrane fusion.

### The different SDS-resistant abilities of the truncated SNARE complex

**Figure 4:**
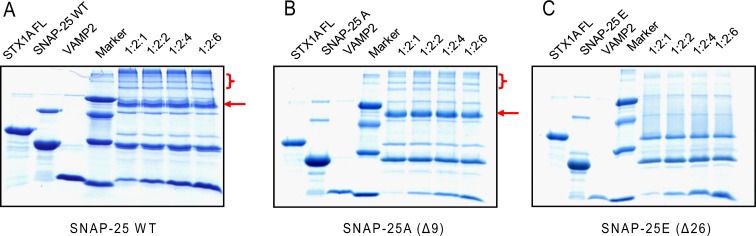
The SDS-resistant ability of the truncated SNARE complex. Four groups of different molecular ratio of one time full-length syntaxin 1A mixes with two times wild-type or truncated SNAP-25 and various soluble VAMP2 and runs the SDS-PAGE without boiling. The markers, from top to bottom, are 66, 45, 29 and 14.2 kDa. (A) Wild-type SNAP-25 with syntaxin 1A and soluble VAMP2, (B) BoNT/A truncated SNAP-25A (Δ9), (C) BoNT/E truncated SNAP-25E (Δ26). The red arrows indicate the position of the SNARE complex; the red brackets indicate the higher order of SNARE complexes due to SNAP-25 swapping.

Former studies show that SNARE proteins can form a very stable complex that is resistant to SDS denaturation and high temperatures (>90 °C) (Hayashi et al., 1995; Wei et al., 2000). Therefore, we compared the SDS resistant property of different SNARE complexes, composed of truncated SNAP-25, syntaxin 1A and VAMP2 (Fig. 4). We mixed syntaxin 1A and wild-type SNAP-25, or truncated SNAP-25A (Δ9) or SNAP-25E (Δ26) at the molar ratio of 1:2, and with four different amounts of soluble VAMP2 (1-, 2-, 4-, 6-fold of syntaxin 1A) at room temperature for one hour, and ran the SDS-PAGE gel without boiling. As expected, the wild-type SNAP-25 formed the SDS-resistant complex with the other two SNARE proteins (Fig. 4A, red arrow). We also found that there were many high molecular weight complexes besides the monomeric SNARE complex, which may be due to the aggregation of SNARE complexes by SNAP-25 swapping (Fig. 4A, red bracket). SNAP-25A (Δ9) formed a SDS-resistant mono-complex and small amounts of high molecular weight complexes (Fig. 4B), while SNAP-25E (Δ26) did not form any SDS-resistant complex (Fig. 4C). Therefore, these results suggest that the SNARE complexes composed of the truncated SNAP-25 display a low stability to the SDS detergent.

### The fusion activity of the truncated SNAP-25 mutants

**Figure 5:**
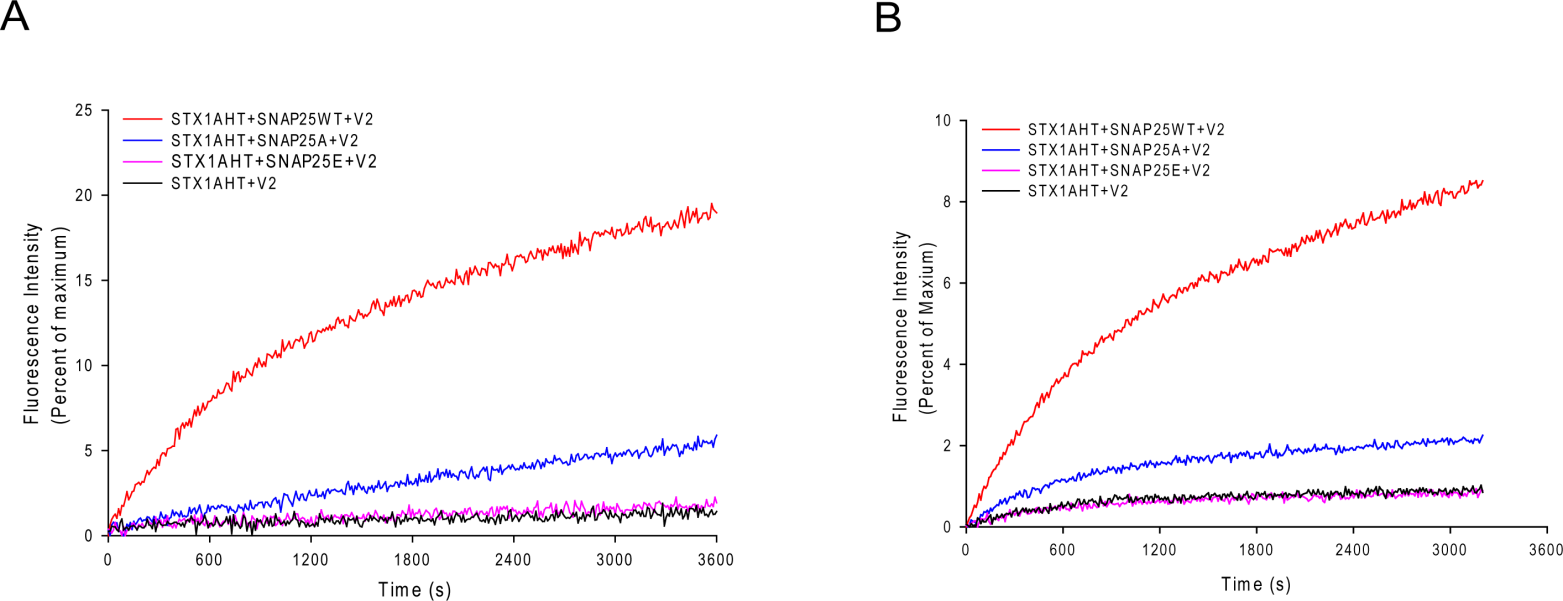
Lipid mixing assay for the truncated SNARE complex. Fluorescence changes normalized against the maximum fluorescence intensity (MFI) are shown for the lipid: protein molar ratio of 100:1 (A) and 200:1 (B). Wild-type SNAP-25 and the truncated SNAP-25A (Δ9) and SNAP-25E (Δ26) are used in the proteoliposome fusion assay. The control runs with the v-SNARE vesicles and the t-SNARE vesicles without the SNAP-25 (black curve).

The less stable truncated SNARE complexes, due to the truncated SNAP-25, may decrease their fusion activity. To test this idea, we used the fluorescence lipid mixing assay to detect the function of the truncated SNAP-25. As Figs. 5A and 5B show, the truncated SNAP-25E (Δ26) formed SNARE complex totally abolished the function to induce membrane fusion, just as in the case of negative control, which has no SNAP-25. The SNAP-25A (Δ9) mutant had only about 6% of maximum fluorescence intensity (MFI), while the wild-type SNAP-25 had about 20% of MFI. So, it is clear that the truncated SNAP-25A lost most of its function, although it could still form the SDS-resistant SNARE complex (Fig. 4B). Our lipid mixing results were also in agreement with the *in vivo* studies, which showed the same tendency of different membrane fusion activities in neurotoxins treated spinal cord cell cultures (Bergey, Bigalke & Nelson, 1987; Williamson, Fitzgerald & Neale, 1992).

### The local structure of the truncated SNARE complex

**Figure 6:**
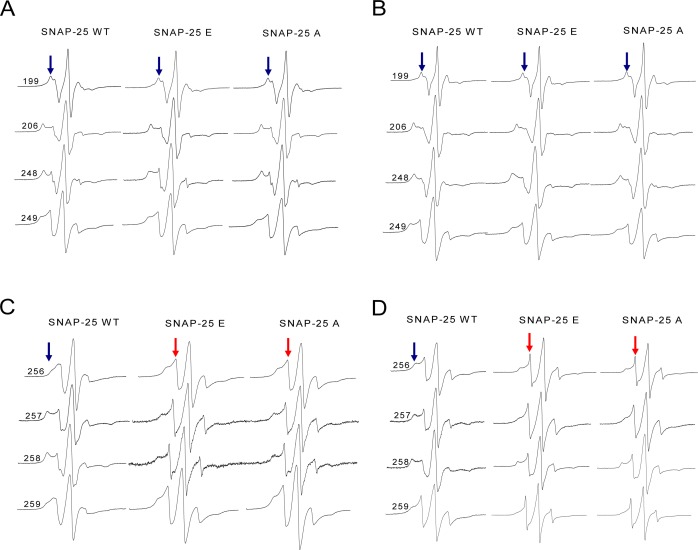
EPR spectra of the spin-labeled full-length syntaxin 1A mutants involved in the wild-type or truncated SNAP-25 and soluble VAMP2 ternary complex. (A) and (B) EPR spectra for spin-labeled positions at 199, 206, 248 and 249 of syntaxin 1A in the ternary SNARE complex. These four positions correspond to the upstream of SNAP-25 SN2 neurotoxins cleavage sites. (A) is the ternary complex in the detergent, (B) is in the membrane. (C) and (D) EPR spectra for positions at 256, 257, 258 and 259 of syntaxin 1A in the ternary SNARE complex. These four positions are the last four amino acids of the syntaxin 1A SNARE motif. (C) is the ternary complex in the detergent, (D) is in the membrane. The mobile and immobile components are indicated in red and blue arrows, respectively.

We are very interested in exploring why there was such a big difference between the wild-type and truncated SNARE complexes. Therefore, we employed CW-EPR spectroscopy to investigate the local conformational changes of the truncated ternary SNARE complex. The spin-labeled syntaxin 1A proteins were incubated with soluble VAMP2 and the truncated SNAP-25 mutants, and then formed ternary complexes. We selected four positions in syntaxin 1A, corresponding to the upstream positions of SNAP-25 neurotoxins cleavage sites (N-terminus). As Figs. 6A and 6B show, in the N-terminal SNARE motif of syntaxin 1A, the spectra were all very broad, both for the wild-type and the truncated SNARE complexes. This suggests that SNARE proteins form the complete coiled coil regardless of the kind of truncated SNAP-25 involved. Since the BoNT/A and BoNT/E cleavage sites were both at a distance from these spin-labeled positions, the truncated SNAP-25 did not disrupt the SNARE core formation.

However, when the syntaxin 1A spin-labeled positions were changed to the last four positions of its SNARE motif (C-terminus), the EPR spectra became much sharper (Figs. 6C and 6D), which reflected the fast motion of nitroxide and indicated that this region is largely unstructured. So, our structural data show that the truncated SNAP-25 mutants induced the partially free conformation of the SNARE core complex in the C-terminus. This tendency was much more obvious when the ternary complex was reconstituted in the membrane, indicating the function of the membrane in the arrangement of the SNARE complex.

## Discussion

Neurotransmitters release requires the complete assembly of three SNAREs, namely syntaxin 1A and SNAP-25 on the target plasma membrane and synaptobrevin 2 (VAMP2) on the synaptic vesicles at the late step of Ca^2+^-dependent regulated exocytosis. Regulatory proteins, such as synaptatogmin and complexin, may modulate the structure of the SNARE complex at each step of the assembly pathway to help form the tight ternary SNARE complex, ultimately leading to membrane fusion. Although some studies suggest that SNAREs may not assemble into any intermediate upon the activation of exocytosis (Lang et al., 2002), it is usually thought that SNAREs assemble gradually into the ultimate ternary complex leading to membrane fusion (Jahn & Scheller, 2006). The active 1:1 syntaxin 1A/SNAP-25 binary complex has been found to be stable and clustered on the plasma membrane in chromaffin cells (Rickman et al., 2004). Moreover, the nucleation of the assembly process required all three N-terminal SNARE motifs of syntaxin 1A and SNAP-25 (Kawasaki & Ordway, 2009), but not VAMP2. It was reported that, *in vitro*, high concentrations of syntaxin would form the 2:1 binary complex with SNAP-25, in which the additional syntaxin occupied the position of VAMP2 in the ternary complex (Zhang et al., 2002). However, our EPR data reveal that the C-terminal of SNAP-25 SN2 in the binary complex is totally free compared to the corresponding positions of the C-terminal of SNAP-25 SN1, which indicates that the 1:1 t-SNAREs binary complex is the dominant form in our pull-down experiment.

In our EPR study, we also detected sharp components at the C-terminal of SNAP-25 when adding soluble VAMP2 to form the *trans*-SNARE ternary complex. This may be due to the tendency of the SNARE complex to fray at the membrane-proximal region, which is close to the initial fusion pore. Another possible scenario is when the SNARE proteins cannot proceed to the tight C-terminal zippering and the complete SNARE assembly needs other regulatory proteins to induce initial fusion pore opening. Complexin and synaptotagmin are two good candidates to play this role (Tang et al., 2006). The N-terminal of complexin may bind to the juxta-membranous sequence of SNAREs to transfer the force from the SNARE complexes to the membranes in fusion (Maximov et al., 2009). The late step also involved Ca^2+^-stimulated manner. Synagtotagmin, a major calcium sensor in synapses, conducts conformational changes after the influx of Ca^2+^, binding to SNAREs and membrane lipids simultaneously and leading to the fast opening of fusion pores (Lee et al., 2010). It has also been shown that BoNT/A treatment essentially increases the Ca^2+^ concentration required to activate exocytosis (Gerona et al., 2000), which indicates that the C-terminus of SNAP-25 mediates Ca^2+^-dependent interactions between synaptotagmin and SNAP-25.

Recently, it has been reported that the C-terminal truncated SNAP-25(Δ9) less-tight complex displays a smaller amperometric foot current, reduces fusion pore conductances, and lowers fusion pore expansion rates in chromaffin cells (Fang et al., 2008). Cleavage by neurotoxins may destabilize the four-helical bundle of the synaptic fusion complex in the C-terminal region, and may disrupt the ability of the complex to join membranes. Our EPR data provide the structural basis for the less-tight zippering at the C-terminal of SNAP-25. Based on the single-vesicle lipid and content mixing results (Y Ishitsuka, 2010, unpublished data), we found that the truncated SNARE complexes already displayed different fusion activities before fusion pore opening, and that the conformational change in the assembled SNARE complex leads to different structures of the initial fusion pore. Considering the distinct fusion activity between SNAP-25A and SNAP-25E, we propose that the sequence between the cleavage sites of BoNT/A and BoNT/E (SNAP-25 Ile^181^–Gln^197^) is important for the stability of SNARE complex and/or its interactions with regulatory proteins.
